# Female Sexual Dysfunction in Women with Non-Malignant Cervical Diseases: A Study from an Urban Chinese Sample

**DOI:** 10.1371/journal.pone.0141004

**Published:** 2015-10-16

**Authors:** Jiehua Ma, Yanjing Kan, Aixia Zhang, Yu Lei, Bin Yang, Ping Li, Lianjun Pan

**Affiliations:** 1 State Key Laboratory of Reproductive Medicine, Department of Reproductive Health, Nanjing Maternity and Child Health Care Hospital affiliated with Nanjing Medical University, Nanjing, Jiangsu, China; 2 State Key Laboratory of Reproductive Medicine, Department of Cervical Care, Nanjing Maternity and Child Health Care Hospital affiliated with Nanjing Medical University, Nanjing, Jiangsu, China; 3 State Key Laboratory of Reproductive Medicine, Department of Nursing, Nanjing Maternity and Child Health Care Hospital affiliated with Nanjing Medical University, Nanjing, Jiangsu, China; 4 Department of Reproductive genetic, The Fourth People's Hospital, Zhenjiang, 212001, China; 5 State Key Laboratory of Reproductive Medicine, Department of Gynecological Endocrinology, Nanjing Maternity and Child Health Care Hospital affiliated with Nanjing Medical University, Nanjing, Jiangsu, China; Shanghai Jiao Tong University School of Medicine, CHINA

## Abstract

Non-malignant cervical diseases are common causes of disease among women worldwide. Although many studies have focused on sexual function in women with cervical cancer, little is known about the prevalence of female sexual dysfunction and its risk factors in women with non-malignant cervical diseases. The present study aims to assess sexual function in Chinese women with non-malignant cervical diseases and to identify potential risk factors for these diseases. A cross-sectional hospital-based survey was conducted in Nanjing, China. The Chinese version of the Female Sexual Function Index (CVFSFI) was used to evaluate sexual function. Three hundred three women who had been diagnosed with at least one non-malignant cervical disease and 293 healthy women were recruited from Nanjing Maternity and Child Health Hospital of Nanjing Medical University. We found that women with non-malignant cervical diseases had a significantly higher prevalence of female sexual dysfunction (FSD) (51.8% vs. 34.8%), low desire (43.2% vs. 26.3%), arousal disorder (41.6% vs. 28.3%), and lubrication disorder (51.2% vs. 36.9%) compared with the control group. Cervicitis and cervical intraepithelial neoplasia (CIN) were found to be independent risk factors for FSD. Our study indicates that women with cervicitis and CIN are at a high risk for FSD and deserve focused initial and follow-up management.

## Introduction

Female sexual dysfunction (FSD) is a common disorder among Chinese women, and it is complicated by and associated with multiple factors, including age, menstrual status, mental health and interpersonal relations [1]. Many studies have focused on the sexual function of women with cervical cancer; however, few reports have investigated the influence of non-malignant cervical diseases on female sexuality [2–4]. Non-malignant cervical diseases are among the most common diseases of women, with a prevalence of morbidity of approximately 35.0% in China [5–6]. Outpatient non-malignant cervical diseases typically comprise cervicitis, cervical intraepithelial neoplasia (CIN), cervical polyps, cervical nabothian cysts, cervical hypertrophy, submucosal uterine fibroids and cervical genital warts. Clinical manifestations usually include mucopurulent discharge from the cervix, cervical friability (easy bleeding from the cervix with passage of a swab) and cervical ectopy [7]. Non-malignant cervical diseases can lead to infertility, miscarriage, and cervical cancer and can have profound social and psychological impacts on a patient’s overall quality of life [8–10]. Gungor et al. indicated that vaginal discharge impacts female sexual function [11]. These studies suggest that non-malignant cervical disease may increase the negative effects of FSD. However, little is known about the sexual function of women with non-malignant cervical diseases; this may be due to the diversity of non-malignant cervical diseases.

In our previous studies, we established clinical cutoff scores for the CVFSFI [12]. The objective of this cross sectional study was to compare the sexual function of Chinese women with non-malignant cervical diseases to a control group and to identify the risk factors for FSD among these women.

## Materials and Methods

### Study Population

This was a cross-sectional survey conducted at Nanjing Maternity and Child Health Hospital of Nanjing Medical University. Our cervical clinic performs screening for cervical disease, which is part of a routine physical examination in departments of gynecology in China. Between May 2012 and December 2013, all women who attended our cervical clinic for regular physical examinations and fulfilled the inclusion criteria, which included women with or without a history of cervical disease, were invited to participate in this study. The inclusion criteria were as follows: junior high school or higher education, regular sexual life, adult Han Chinese women, and inhabitants of Nanjing. The validation of the CVFSFI in 2011 did not involve ethnic differences; however, cultural traditions of different ethnic groups are discrepant, which may affect attitudes towards sex [13]. In addition, most of the residents of Nanjing are Han Chinese; other ethnic groups are rare. Therefore, we only investigated Han Chinese women. The exclusion criteria were as follows: unwillingness to participate, unable to read or understand the study, lack of sexual history, pregnant or breastfeeding, suffering from a critical illness, and partner with sexual dysfunction. Some of the exclusion criteria involve intimate and taboo topics in China. In order to make the selection process more simple and feasible, the information of pregnant or breastfeeding, suffering from a critical illness, and partner with sexual dysfunction were collected in the questionnaire and the exclusion was done after finishing the questionnaire. Sample size was determined with a power analysis using preliminary data obtained in our hospital with the following assumptions: α of 0.05 (two-tailed), power of 90.0%, non-malignant cervical disease group proportion of 0.5, control group proportion of 0.35. Based on these assumptions, a minimum of 144 patients in each group was needed.

### Ethics Statement

All participants provided written informed consent, and the study was approved by the Institutional Ethics Committee of Nanjing Maternity and Child Health Care Hospital affiliated with Nanjing Medical University (Process n.2012/3).

### Measures

A trained gynecologist was available to help participants with any survey difficulties, and participants were allowed to complete the questionnaire alone in a dedicated room. Women who participated in this study were classified according to whether they had any cervical lesion. Cervical health status was assessed by cervical specialists after cervical examination. The cervical examinations include cervical smears, thinprep liquid-based cytology test (TCT), microbiology and colposcopy. Malignant disease was ruled out by TCT. This was a double-blind survey, and the survey was completed before diagnosis of cervical disease was made. Women were examined after completing the survey, and the clinicians making the diagnosis were not aware of the survey results.

The health status of the patient and her partner were classified as healthy (having no disease and feeling full of energy), average (having some discomfort, such as cold, but which does not cause distress), and poor (having chronic diseases and feeling distress). The female sexual function index (FSFI) is a 19-item, self-administered screening questionnaire for assessing female sexual function. Questions are grouped into the following six domains: desire, arousal, lubrication, orgasm, satisfaction and pain. The FSFI full score is obtained by adding the six domain scores. A higher FSFI score represents better sexual function [14]. This study used the CVFSFI, which was validated in 2011 and has been utilized in several studies [2, 12, 13, 15, 16]. We established clinical cutoff scores for the CVFSFI in our previous study [12]. A total score equal to or less than 23.45 is the cutoff value to define FSD. Cutoff scores for the domains are as follows: define FSD. Cutoff scores ubrication, orgasm, satisfaction and pain. The FSFI full score is obtained 3.8, sexual pain.

### Statistical Analyses

All statistical analyses were conducted using SPSS version 17.0(SPSS Inc., Chicago, IL, USA). An independent-samples t-test was used to calculate the domain scores of FSD. The chi-square test was used to compare the prevalence of FSD between the groups. To determine independent risk factors for FSD, we performed binary logistic regression analysis. A *p*-value less than 0.05 was considered to be statistically significant.

## Results

A total of 873 sexually active women who met the inclusion criteria were asked to participate; of these, 738 agreed, and 135 refused. Of the 738 women who agreed to participate, 69 failed to complete the questionnaires, and 73 were excluded. The 73 patients were excluded due to the following exclusion criteria: pregnant or breastfeeding, suffering from a critical illness, and a partner with sexual dysfunction. Thus, 596 questionnaires were analyzed. Table 1 lists all questionnaire variables and the corresponding response options. Women were classified depending on the presence or absence of non-malignant cervical disease. Three hundred three women had at least one non-malignant cervical disease at the time of the study, and thus comprised group A, and 293 had no cervical disease, and thus comprised group.

**Table 1:** General characteristics of the study population.

Characteristics	N(%)	Characteristics	N(%)
**Age**		**Health status**	
< 30 years	89 (14.9)	Healthy	320 (53.7)
30–39 years	246 (41.3)	Average	252 (42.3)
40–49 years	216 (36.2)	Poor	24 (4.0)
50–55 years	45 (7.5)	**Health status of partner**	
**Marriage**		Healthy	445 (74.4)
Married	565 (94.8)	Average	146 (24.5)
Unmarried but have a regular partner	29(4.9)	Poor	5 (0.8)
Divorced but have a regular partner	2(0.3)	**Sexual status of partner**	
**Have a child**		good	376 (63.1)
Yes	509 (85.4)	Average	220 (36.9)
No	87 (14.6)	**History of non-malignant cervical disease**	
**Labor mode**		unknown	24.7(147)
Natural	409(68.6)	Yes	18.8 (112)
Dystocia	9 (1.5)	No	56.5 (337)
Cesarean section	91 (15.3)	**Cervical lesion**	
**Contraception**		None	293 (49.2)
Intrauterine device	218 (36.6)	cervicitis	159 (26.7)
Condom	219 (36.7)	CIN	121 (20.3)
Oral contraceptives	9 (1.5)	Cervical polyps	50 (8.4)
Ligation of fallopian tubes	28 (4.7)	Nabothian cyst	15 (2.5)
Others	122 (20.5)	cervical hypertrophy	38 (6.4)
		cervical submucosal uterine fibroids	5 (0.8)
		cervical genital warts	3 (0.5)

The total FSFI score and the domain scores of non-malignant cervical diseases and controls are shown in Table 2. Statistical analysis was not performed among participants with cervical submucosal uterine fibroids or cervical genital warts due to the small number of women with these conditions. The analysis revealed no significant difference in the FSFI scores for orgasm or pain. However, women with non-malignant cervical diseases had a significantly lower FSFI scores for desire, arousal, lubrication, and total FSD score.

**Table 2:** FSFI scores of female sexual desire, arousal, lubrication, orgasm, satisfaction, and pain (n = 596).

Group	n	Desire	Arousal	Lubrication	Orgasm	Satisfaction	Pain	Total score
**Cervicitis**								
Yes	159	2.72±0.60*	3.28±0.59*	4.08±0.71*	4.40±0.98	4.09±0.79*	4.70±0.95	23.26±2.91*
No	437	3.06±0.74	3.42±0.71	4.25±0.89	4.30±0.87	4.44±0.79	4.63±0.90	24.09±3.38
**CIN**								
Yes	121	2.75±0.55*	3.22±0.52*	4.03±0.66*	4.27±0.84	4.16±0.70*	4.70±0.81	23.13±2.34*
No	475	3.02±0.75	3.43±0.71	4.25±0.88	4.33±0.92	4.39±0.82	4.63±0.94	24.06±3.46
**Cervical polyps**								
Yes	50	2.74±0.60*	3.19±0.64*	3.91±0.81*	4.34±1.03	4.22±0.82	4.68±0.88	23.07±3.30
No	546	2.98±0.73	3.40±0.68	4.24±0.84	4.32±0.89	4.36±0.80	4.64±0.92	23.95±3.27
**Nabothian cyst**								
Yes	15	2.92±0.45	3.32±0.42	3.76±0.91*	4.48±1.05	4.13±0.85	4.67±1.02	23.28±3.40
No	581	2.97±0.73	3.39±0.69	4.22±0.84	4.32±0.90	4.35±0.80	4.65±0.91	23.89±3.28
**Cervical hypertrophy**								
Yes	38	2.78±0.58	3.27±0.68	3.89±0.83*	4.44±1.05	4.09±0.88*	4.71±0.97	23.18±3.51
No	558	2.98±0.73	3.39±0.68	4.23±0.84	4.31±0.89	4.36±0.80	4.64±0.91	23.92±3.26
**Cervical submucosal uterine fibroids (No statistical analysis)**								
Yes	5	2.40±0.42	3.36±0.78	4.02±0.75	3.68±0.87	4.08±0.52	4.00±1.47	21.54±3.70
No	591	2.97±0.72	3.38±0.68	4.21±0.85	4.33±0.90	4.35±0.81	4.65±0.91	23.89±3.27
**Cervical genital warts**								
**(No statistical analysis)**								
Yes	3	2.00±0.35	3.10±0.46	4.20±0.60	4.13±1.22	4.80±0.80	5.20±0.40	23.43±1.64
No	593	2.97±0.72	3.39±0.68	4.21±0.85	4.33±0.90	4.34±0.80	4.64±0.91	23.88±3.29
**non-malignant cervical diseases**								
Yes	303	2.72±0.59*	3.24±0.56*	3.99±0.74*	4.33±0.93	4.13±0.76*	4.67±0.90	23.10±2.84*
No	293	3.22±0.76	3.53±0.76	4.43±0.90	4.31±0.87	4.56±0.79	4.62±0.92	24.67±3.51
**Total**	596	2.97±0.72	3.38±0.68	4.20±0.85	4.32±0.90	4.35±0.80	4.65±0.91	23.87±3.30

*: P<0.05, compared to the group answering “No”.

FSFI: Female Sexual Function Index. CIN: cervical intraepithelial neoplasia.

Using the cutoff scores of the CVFSFI, we investigated the prevalence of FSD among the groups. Compared to the control group, women with non-malignant cervical diseases had a significantly higher prevalence of FSD (51.8% vs 34.8%, *p*<0.01), low desire (43.2% vs 26.3%, *p*<0.01), arousal disorder (41.6% vs 28.3%, *p*<0.01), and lubrication disorder (51.2% vs 36.9%, *p*<0.01), but there were no significant differences between the groups for orgasm disorder (*p* = 0.528) or sexual pain (*p* = 0.308) (Table 3). The prevalence of low desire (42.8% vs 32.0% for cervicitis, *p* = 0.015; 42.1% vs 33.1% for CIN, *p* = 0.061) and FSD (49.7% vs 41.2% for cervicitis, *p* = 0.064; 66.7% vs 43.3% for CIN, *p* = 0.011) was much higher in women with cervicitis and CIN than in controls. In addition, women with CIN had a significantly higher prevalence of arousal disorder (44.6% vs 32.6%, *p* = 0.014). Significant differences were also found for some other non-malignant cervical diseases; for example, women with cervical polyps had a higher prevalence of lubrication disorder (60.0% vs 42.7%, *p* = 0.018) than controls. However, cervical polyps, cervical nabothian cysts, cervical hypertrophy, cervical submucosal uterine fibroids, and cervical genital warts were not entered into the regression model in logistic regression analysis.

**Table 3:** Prevalence of FSD determined by CVFSFI cutoff values among groups (n = 596, %).

Group	n	Low desire	Arousal disorder	Lubrication disorder	Orgasm disorder	Sexual pain	FSD
**Cervicitis**							
Yes	159	42.8	37.1	47.8	26.4	19.5	49.7
No	437	32.0	34.3	42.8	30.0	20.8	41.2
*P* value		0.015	0.529	0.276	0.397	0.723	0.064
**CIN**							
Yes	121	42.1	44.6	48.8	32.2	15.7	66.7
No	475	33.1	32.6	42.9	28.2	21.7	43.3
*P* value		0.061	0.014	0.250	0.382	0.145	0.011
**Cervical polyps**							
Yes	50	40.0	48.0	60.0	34.0	16.0	52.0
No	546	34.4	33.9	42.7	28.6	20.9	42.7
*P* value		0.429	0.045	0.018	0.418	0.413	0.203
**Cervical nabothian cyst**							
Yes	15	33.3	20.0	53.3	26.7	33.3	53.3
No	581	34.9	35.5	43.9	29.1	20.1	43.2
*P* value		0.897	0.215	0.467	0.838	0.211	0.434
**Cervical hypertrophy**							
Yes	38	44.7	36.8	50.0	28.9	21.1	47.4
No	558	34.2	34.9	43.7	29.0	20.4	43.2
*P* value		0.189	0.813	0.451	0.991	0.927	0.615
**Cervical submucosal uterine fibroids**							
**(No statistical analysis)**							
Yes	5	80.0	60.0	60.0	40.0	40.0	60.0
No	591	34.5	34.9	44.0	28.9	20.3	43.3
**Cervical genital warts**							
**(No statistical analysis)**							
Yes	3	100	100	33.3	33.3	100	66.7
No	593	34.6	34.9	44.2	29.0	20.6	43.3
**non-malignant cervical diseases**							
Yes	303	43.2	41.6	51.2	30.0	18.8	51.8
No	293	26.3	28.3	36.9	28.0	22.2	34.8
*P* value		<0.01	<0.01	<0.01	0.582	0.308	<0.01
**Total**	596	34.9	35.1	44.1	29.0	20.5	43.5

CVFSFI: the Chinese version of the Female Sexual Function Index. CIN: cervical intraepithelial neoplasia.

We also assessed the relationships between female sexual function and non-malignant cervical diseases by correlation analysis. Statistically significant negative correlations were found between non-malignant cervical diseases and FSFI scores in all domains except for orgasm (*p* = 0.848) and pain (*p* = 0.412) (Table 4). Independent risk factors for FSD as calculated by binary logistic regression analysis are shown in Table 5. After adjusting for variables in Table 1, the analysis showed that age, health status, sexual status of the partner, cervicitis and CIN were independent risk factors for FSD.

**Table 4:** Correlation between FSFI scores and non-malignant cervical diseases.

		desire	arousal	lubrication	orgasm	satisfaction	pain	total
**Cervicitis**	Correlation Coefficient	-0.194**	-0.083*	-0.088*	0.064	-0.210**	0.043	-0.107**
	*P* value	<0.01	.044	0.031	0.119	<0.01	0.291	<0.01
**CIN**	Correlation Coefficient	-0.153**	-0.126**	-0.103*	-0.048	-0.142**	0.024	-0.137**
	*P* value	<0.01	<0.01	0.012	0.239	<0.01	0.559	<0.01
**Cervical polyps**	Correlation Coefficient	-0.095*	-0.082*	-0.104*	0.007	-0.037	0.006	-0.067
	*P* value	0.020	0.045	0.011	0.871	0.372	0.889	0.102
**Cervical nabothian cyst**	Correlation Coefficient	-0.012	-0.012	-0.070	0.016	-0.055	0.004	-0.027
	*P* value	0.777	0.772	0.090	0.693	0.182	0.919	0.504
**Cervical hypertrophy**	Correlation Coefficient	-0.068	-0.028	-0.083*	0.042	-0.080	0.017	-0.034
	*P* value	0.096	0.488	0.042	0.309	0.051	0.671	0.411
**Cervical submucosal uterine fibroids**	Correlation Coefficient	-0.082*	-0.008	-0.020	-0.062	-0.045	-0.035	-0.055
	*P* value	0.044	0.844	0.623	0.130	0.271	0.391	0.182
**Cervical genital warts**	Correlation Coefficient	-0.099*	-0.036	0.001	-0.010	0.040	0.051	-0.016
	*P* value	0.016	0.376	0.978	0.810	0.332	0.217	0.702
**Non-malignant cervical diseases**	Correlation Coefficient	-0.340**	-0.213**	-0.238**	0.008	-0.299**	0.034	-0.242**
	*P* value	<0.01	<0.01	<0.01	0.848	<0.01	0.412	<0.01

**: correlation is significant at the 0.01 level.

*: correlation is significant at the 0.05 level.

FSFI: Female Sexual Function Index. CIN: cervical intraepithelial neoplasia.

**Table 5:** Risk factors for FSD: a binary logistic regression analysis.

				95% CI	
Exposure variables	B	*P*	OR	Lower	Upper
Health status	0.372	0.019	1.451	1.064	1.978
Sexual status of partner	0.938	<0.01	2.555	1.763	3.702
Cervicitis	0.458	0.020	1.581	1.074	2.327
Age	0.328	<0.01	1.388	1.119	1.722
CIN	0.521	0.016	1.684	1.104	2.570

FSD: female sexual dysfunction. CIN: cervical intraepithelial neoplasia.

## Discussion

FSD is multifactorial and encompasses a wide range of physiological and emotional processes [17]. Studies have shown a high incidence of FSD among women with cervical cancer [3]. However, do non-malignant cervical diseases affect female sexual function? Few reports on this subject can be found. Worldwide, non-malignant diseases are prevalent and complicated. In the presence of non-malignant cervical diseases, the negative effects of FSD, such as psychological disorders, social problems, and high divorce rate, are exacerbated. Therefore, we performed this hospital-based study to conduct a comprehensive assessment of sexual function among Chinese women with non-malignant cervical diseases.

Our cervical clinic performs screening for cervical disease. The screening is part of a routine physical examination of gynecology in China. The women attending the clinic were aware that the clinic specializes in cervical conditions, which may affect the prevalence of the conditions found and may also affect the findings of FSD. However, we believe that any such influence is limited due to the double-blind design. Despite this, further research with a larger sample size and a more strictly selected population is needed.

We utilized the CVFSFI, a well-validated and widely used instrument to evaluate female sexual function [18, 19]. The results showed significant between-group differences in FSFI scores for desire, arousal, lubrication, satisfaction and overall FSD. Diagnostic points established in our previous study were used to distinguish between Chinese women with or without sexual dysfunction. The demarcation of satisfaction disorder was not included in this study due to a lack of diagnostic criteria [12].

The survey suggested that 51.8% of women with non-malignant cervical disease suffer from sexual dysfunction, compared with only 34.8% of the control group, which indicated that non-malignant cervical diseases may affect female sexual function. The prevalence of FSD among the general population of healthy women has been found to be 31.0% to 50.0%, which is consistent with our results [2]. Gynecological examination is not a part of routine physical examinations in China. In our previous study, we found a higher rate (37.6%) of FSD among the general population of women, which might be attributed to an incomplete exclusion of women with non-malignant cervical diseases. Of note in the current study, we found a significant difference in the prevalence of FSD among women with cervicitis and CIN, which was consistent with the results of logistic regression analysis. Significant differences were also found in some other non-malignant cervical diseases, which did not enter the regression model. This is most likely due to the impact of confounding factors.

The finding of a lower prevalence of sexual pain among women with non-malignant cervical diseases (18.8%) compared with the controls (22.2%) is difficult to understand. However, our finding is similar to a survey by Gungor et al., who found that women with abnormal vaginal discharge had better FSFI scores in some domains [11]. Additional studies using a larger group are needed to evaluate the relationship between sexual pain and non-malignant cervical diseases.

Correlation analysis showed that non-malignant cervical diseases were negatively correlated with desire, arousal, lubrication and total score, which correlated with overall FSD score and morbidity differences between the two groups. However, the correlations were weak (correlation coefficients of approximately 0.2), and further research using lager samples is needed. Female sexuality is multifactorial and includes a wide range of emotional and physiological processes. Therefore, the effects of low desire, arousal disorder and lubrication disorder might be diluted or buffered by other factors, effectively reducing their relative impacts.

When analyzing risk factors for FSD using binary logistic regression analysis, we found that age, health status, sexual status of the partner, cervicitis and CIN were independent risk factors for FSD. Age, health status and the sexual status of partner were also identified as risk factors in our previous study [1]. Cervicitis manifests with increased and purulent vaginal discharge accompanied by back pain and abdominal discomfort. Thus, cervicitis might cause physical discomfort and psychological stress, which would influence female sexual function. CIN consists of precancerous lesions which cause stress, anxiety, and depression [20]. As a result, CIN may affect sexual pleasure. However, we found no significant difference in the rate of FSD for different grades of CIN. Thus, women with CIN in our report were not analyzed further in the present analysis.

Some limitations of this study must be noted. First, the study cohort was not population-based but rather hospital-based, which affects the generalizability of these findings to the community. However, a hospital-based study is an appropriate method of investigation considering the conservative traditional Chinese attitude toward sex and the fact that Chinese women are unwilling to discuss sexual issues openly. Third, there are few studies of sexual function among women with non-malignant cervical diseases, thus, we have to make reference and compare some of our results to the female sexual dysfunction found among cervical cancer patients. This seems inappropriate because patients with cervical cancer may have many other reasons for sexual dysfunction, including having undergone surgery and/or chemoradiation to have sexual dysfunction. Finally, the number of patients with specific cervical diseases, such as cervical submucosal uterine fibroids and cervical genital warts was very small. We performed only descriptive analysis of these groups instead of conducting comparisons between them. Thus, studies with larger samples are necessary to elucidate the real impact of non-malignant cervical diseases on female sexual function.

## Conclusion

Women in China with non-malignant cervical diseases have a higher prevalence of female sexual dysfunction. Cervicitis and CIN are independent risk factors for FSD. Further studies with women from diverse backgrounds are needed to understand the epidemiology of the disorder.

## Supporting Information

S1 Data (XLSX)
